# Auditory Pattern Representations Under Conditions of Uncertainty—An ERP Study

**DOI:** 10.3389/fnhum.2021.682820

**Published:** 2021-07-09

**Authors:** Maria Bader, Erich Schröger, Sabine Grimm

**Affiliations:** Cognitive and Biological Psychology, Institute of Psychology—Wilhelm Wundt, Faculty of Life Sciences, Leipzig University, Leipzig, Germany

**Keywords:** auditory processing, P3a, complex sound patterns, event-related potentials, mismatch negativity

## Abstract

The auditory system is able to recognize auditory objects and is thought to form predictive models of them even though the acoustic information arriving at our ears is often imperfect, intermixed, or distorted. We investigated implicit regularity extraction for acoustically intact versus disrupted six-tone sound patterns *via* event-related potentials (ERPs). In an exact-repetition condition, identical patterns were repeated; in two distorted-repetition conditions, one randomly chosen segment in each sound pattern was replaced either by white noise or by a wrong pitch. In a roving-standard paradigm, sound patterns were repeated 1–12 times (standards) in a row before a new pattern (deviant) occurred. The participants were not informed about the roving rule and had to detect rarely occurring loudness changes. Behavioral detectability of pattern changes was assessed in a subsequent behavioral task. Pattern changes (standard vs. deviant) elicited mismatch negativity (MMN) and P3a, and were behaviorally detected above the chance level in all conditions, suggesting that the auditory system extracts regularities despite distortions in the acoustic input. However, MMN and P3a amplitude were decreased by distortions. At the level of MMN, both types of distortions caused similar impairments, suggesting that auditory regularity extraction is largely determined by the stimulus statistics of matching information. At the level of P3a, wrong-pitch distortions caused larger decreases than white-noise distortions. Wrong-pitch distortions likely prevented the engagement of restoration mechanisms and the segregation of disrupted from true pattern segments, causing stronger informational interference with the relevant pattern information.

## Introduction

Acoustic information, which arrives at ours ears and informs us about objects in the outer world, is often imperfect. Parts of the relevant object information might be obscured by extraneous noise from concurrent auditory sources, for instance, from some sudden, interfering background sounds like a honking car or a barking dog. In other instances, parts of the relevant auditory objects can be missing. Furthermore, particularly in the domains of speech and music, mistakes in production might lead to imperfect recurrences of the same object, for instance, when a familiar melody is played with a missing or a wrong note or a word is uttered with an incorrect phoneme. Such occurrences can intermix with or distort the acoustic input we are currently paying attention to, and might impede its intelligibility.

Our ability to deal with such distortions and to interpret the acoustic environment, even if it is degraded to a certain degree, is a remarkable asset. One should note that, on the one hand, our auditory system is able to detect even slight variations in the acoustic input. Previous studies found that the auditory system is sensitive to very subtle acoustic changes (e.g., in sound frequency), particularly if the respective varying sound event occurs after a row of invariant repetitions (Sams et al., 1985; Winkler et al., 1990). On the other hand, our brain must be able to tolerate variation to some extent. That is, we need to neglect input variation that is irrelevant for the current task or we need to compensate for missing or distorted information. Usually, we are well able to maintain a stable representation of objects in the environment, even if we encounter them occasionally in a degraded form. In fact, mechanisms of perceptual prediction and restoration help us to fill in and reconstruct occluded or obscured information. This occurs not only in the visual world (e.g., in the case of the blind spot; Walls, 1954; Ramachandran, 1992; De Weerd, 2006; Spillmann et al., 2006) but also in the auditory domain. Studies on the continuity illusion show that a tone or a word containing a short gap may be perceived as continuous if the gap is filled with noise. The listener then perceives the missing information, suggesting that the auditory system predicts and interpolates through the absent information (Warren et al., 1997; Micheyl et al., 2003; Riecke et al., 2007; Shahin et al., 2009; Bendixen et al., 2014).

Especially in speech and music research, perceptual restoration and filling-in processes are attributed to top-down influences (DeWitt and Samuel, 1990; Shinn-Cunningham and Wang, 2008). When phonemes are replaced with white noise, a word utterance can still seem intact, and, oftentimes, listeners cannot say which part of the uttered sentence was missing (Warren and Warren, 1970). Although this effect is clearly influenced by knowledge and experience, part of the phenomena might occur pre-attentively on lower levels of processing (Micheyl et al., 2003; Riecke et al., 2007).

Electrophysiological markers like the mismatch negativity (MMN) can serve as an index for such implicit and automatic compensatory mechanisms of early processing stages (Micheyl et al., 2003). The MMN is elicited by sounds violating a detected regularity in a sequence of sounds, for example, when a tone differing in pitch (deviant) is presented following a sequence of tones with identical pitch (standards). MMN reflects the process of deviance detection, and its presence indirectly implies that the regularity inherent in the standard tones has been encoded (Winkler, 2007). Although early accounts interpreted the MMN as the outcome of a retrospective comparison process between regularity representations and the incoming deviant sound, newer accounts emphasize that regularity representations are part of an internal model, prospectively generating predictions about future input (Wacongne et al., 2012; Bendixen et al., 2014; Winkler and Schröger, 2015). Previous studies showed that predictions and regularity representations of standards build up even in the absence of exact repetitions of a stimulus, for instance, when using abstract regularities (Tervaniemi et al., 2000; Brattico et al., 2006; Bendixen and Schröger, 2008; Bader et al., 2017) or in the presence of noise that degrades the physical information (Muller-Gass et al., 2001; Micheyl et al., 2003; Kozou et al., 2005). All these studies demonstrate the tolerance of the MMN systems to a considerable amount of variability in the sequence of standard sounds, including cases of imperfect repetitions of standard sounds.

These findings also align with studies on auditory object segregation. For example, McDermott et al. (2011) showed that the auditory system quickly recognizes invariant patterns, even when they are embedded in a changing acoustic background of competing sounds. The authors suggest a mechanism of cross-correlating dynamic spectrotemporal input patterns, which filters for invariances between different occurrences of the same auditory event (despite its being mixed with background noise). Nevertheless, pattern recognition is impaired for sound patterns that are not identically repeated within the mixtures of changing backgrounds. In event-related potentials (ERP) studies, deviant sounds elicit an MMN of decreased amplitude and increased latency in cases where regularity formation is impeded by abstract variations or noise masking (Muller-Gass et al., 2001; Niemitalo-Haapola et al., 2015; Bader et al., 2017).

The MMN component can be followed by a P3a component distributed over fronto-central scalp regions. Typically, P3a is interpreted as signaling an involuntary attentional switch from a primary task to the deviant stimulus (Squires et al., 1975; Escera et al., 2000; Friedman et al., 2001; Wetzel and Schröger, 2007). P3a is sometimes also discussed to reflect a higher level but automatic evaluation of novelty rather than the switch of attention itself (Horvath et al., 2008; Wetzel et al., 2013; Winkler and Schröger, 2015). The P3a amplitude is modulated by cognitive and working memory demands of the task (Berti et al., 2004). P3a amplitude and latency can be modulated by experimental manipulations affecting salience, such as in the presence of abstract variations (Bader et al., 2017) and quality degradations of the regularity inherent in the standards, for example, by added noise, interruptions, or frequency distortions (Micheyl et al., 2003; Bader et al., 2017; Uhrig et al., 2017). That is, P3a amplitude might be decreased and latency increased if deviants violate a regularity that is not defined by exact sound or pattern repetitions, for instance, in the case of a new melodic pattern occasionally occurring among pattern repetitions in a transposed form (Bader et al., 2017).

In the current study, we investigated the early phase of implicit auditory pattern learning. The presented sounds were attended; however, the pattern regularity rule was irrelevant for the task at hand. Thus, learning occurred incidentally rather than intentionally (Perruchet and Pacton, 2006). During the experiment, a standard six-tone pattern is either repeated identically to the listeners or repeated, containing distortions, which leave only 5/6 of the standard pattern intact during each presentation. In particular, the three experimental conditions included (1) an exact-repetition condition (exact) in which sound patterns within a train were perfect repetitions without any distortion; (2) a white-noise-distortion condition (wn) in which one tonal segment of the repeating sound pattern was replaced randomly by Gaussian noise, and (3) a wrong-pitch-distortion condition (wp) in which one tonal segment of the repeating sound pattern underwent a random shift in pitch. The respective distortions could affect a different tonal segment in each pattern presentation; the same segment would be intact in the next pattern presentation. Importantly, we were not measuring the ERPs to the distortions in every trial but, rather, the ERPs to occurrences of a completely new pattern, whether or not the preceding pattern learning happened with or without distortions. If the buildup of a pattern representation is (at least partly) robust against pattern variance in complex auditory stimuli, we hypothesize that MMN and P3a will be elicited in conditions with and without distortions.

However, given that a lower quantity of unambiguous information guides the initial learning process, one might expect poorer pattern representation from distorted standard sounds as indicated by decreased MMN and P3a amplitude and possible latency delays. Given that an equal portion (five out of six tones) was unambiguously informative about the identity of the standard pattern in both distortion conditions, one might expect to find similar impairments in the two distortion conditions. Nevertheless, there are certainly qualitative differences between white noise and wrong pitch distortions. Noise contains the frequencies of the “true” pattern segment and can involve filling-in or restoration processes, once an internal representation of the standard pattern has been formed; whereas wrong pitch distortions might interfere with the formation of a pattern representation. Therefore, differential effects on MMN and/or P3a could be expected in the two distortion conditions, particularly with stronger impairments in the wrong-pitch condition. If that is the case, one can conclude that potentially different mechanisms rule the formation of pattern identity representations in the context of white noise or wrong pitch distortions. This would also be compatible with masking studies, showing stronger degrading effects on speech intelligibility when it is masked by similar stimulus material, such as irrelevant speech (as in the case of informational masking), than when it is masked by noise (as in the case of energetic masking) (Cooke et al., 2008; Lidestam et al., 2014).

Furthermore, if we observed such distortion-specific effects already at the level of MMN, this would argue in favor of an impact of our manipulation on relatively early merely perceptual and automatic processing levels. In contrast, if they were confined to the P3a level, this would indicate that distortion processing occurs only at a later stage in the course of more context-dependent novelty evaluation and automatic orienting of attention.

As mentioned above, we expect MMN and P3a elicitation to full pattern changes in all conditions and a possible differentiable effect of the inserted distortions. These effects might also interact with the number of preceding standards. Therefore, the number of standard patterns (with or without distortions) preceding a deviant was varied (1, 2, 3, 6, or 12 standard patterns) in a roving-standard paradigm (Winkler et al., 1996; Bendixen and Schröger, 2008; Garrido et al., 2009). According to previous studies (Cowan et al., 1993; Winkler et al., 1996; Bendixen et al., 2007; Garrido et al., 2008; Bader et al., 2017), we expect to see a growth in MMN (and P3a) amplitude with increasing numbers of preceding standards in all conditions, if the implicit learning mechanisms are robust against the inserted distortions. Likely, both standard and deviant ERPs contribute to the MMN increase with deviant ERPs growing more negative and standard ERPs growing more positive (and *vice versa* for the P3a) as a function of the number of previous standards in a train (Baldeweg et al., 2004; Bendixen et al., 2007; Costa-Faidella et al., 2011; Bader et al., 2017). We will model the emergence and growth, using a logarithmic regression analysis, because regression coefficients can be informative of the time course and the strength of the implicit formation of pattern representations in our three distortion conditions.

## Materials and Methods

### Participants

The experimental protocol was approved by the Ethical Committee of the University of Leipzig and was in accordance with the Code of Ethics of the World Medical Association (Declaration of Helsinki). The participants gave written informed consent before experimental sessions. All the subjects in the experiment participated for credit points or monetary compensation (€8 per hour). Of the 19 healthy subjects (age range: 19–40 years, 18 females) that participated in this research, all reported normal hearing and 17 out of 19 participants were right-handed. All of them were Leipzig University students, and 84% reported to have played a musical instrument for some time (*M* = 6.6 years, *SD* = 4.2 years).

### Materials

As in one of our previous studies (Bader et al., 2017), auditory stimuli were composed of 300-ms sound patterns, consisting each of six concatenated 50-ms segments with randomly chosen fundamental frequencies between 220 and 880 Hz (in 25 semitone steps). Harmonics were added to each fundamental frequency until a cutoff at 6,000 Hz. Starting at 3,000 Hz, tonal segments were modulated by reducing the signal linearly resulting in 0% intensity at 6,000 Hz. For a smoother sound, odd harmonics (uneven positive integer multiples of the fundamental frequency) were additionally attenuated to 20% of their intensity. To prevent loudness differences between segments, intensities were root mean square equalized. Segments included a 5-ms rise and a 5-ms fall time, and there were no gaps introduced between the six segments when concatenating them to a sound pattern. The stimulus onset asynchrony (SOA) between patterns presented in our auditory sequences was set to 650 ms.

### Design and Procedure

Sound patterns were presented in a roving-standard paradigm (Cowan et al., 1993; Winkler et al., 1996; Bendixen et al., 2007; Garrido et al., 2008) with varying train lengths; a randomly generated sound pattern was presented either 1, 2, 3, 6, or 12 times in a sequence before a newly generated pattern occurred, which started a new train of stimuli. In a single block of the experiment, each possible train length occurred 10 times in random order, resulting in 240 pattern presentations per block. As in our previous study (Bader et al., 2017), 10 additional trials of train length 1 were included in each block, in the way that one trial of train length 1 was directly followed by another trial of train length 1. In this context, three pattern changes always occurred in a row. This served to have pattern changes that did not follow a pattern repetition, and it ensured the investigation of memory trace formation, starting with a first pattern presentation. Here, the first pattern change served as a deviant with respect to the previous train, the second served as a “standard” of train length 1, and the third served as a “deviant” of train length 1. This terminology is consistent with the one used for other train lengths but, admittedly, arbitrary, since stimuli of train length 1 do not have an actual history of pattern repetition. Overall, for each train length, a similar number of standard and deviant patterns was available for ERP analysis (Bader et al., 2017).

**FIGURE 1:**
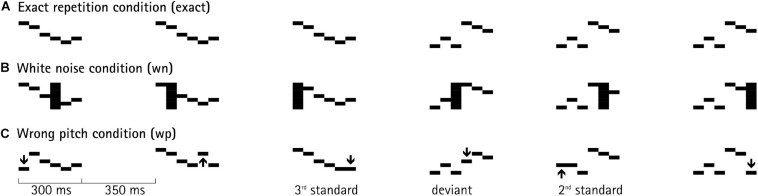
An example of a pattern sequence, in which patterns were presented in a roving-standard paradigm. At the third position, the pattern has been presented three times; before, at the fourth position, a new train started with a new sound pattern (deviant), which, itself, is repeated within that next train. Each deviant corresponds to a first standard of a new train. Each pattern was composed of six concatenated 50-ms segments, differing in fundamental frequency (black horizontal bars). SOA (stimulus onset asynchrony) was set to 650 ms (= 300-ms pattern duration + 350-ms interstimulus interval). The *top line* **(A)** depicts the identical repetition of a sound pattern within a train in the exact-repetition condition. The *middle line* **(B)** depicts the white-noise-distortion condition, in which a randomly chosen segment of each sound pattern was replaced by white noise, marked with the black vertical bar. The *bottom line* **(C)** depicts the wrong-pitch condition, in which a randomly chosen segment of each sound pattern was replaced by a new segment of randomly chosen new pitch, indicated by the arrow.

The experiment consisted of two sessions with a total of 36 blocks (12 blocks × 3 conditions). In each session, six blocks of each condition were presented in random order. In the exact-repetition condition, patterns were repeated 100% identically within a train. In the white-noise condition, during each presentation of a melodic pattern, one randomly chosen segment out of the six was replaced by a 50-ms snippet of white noise. In the wrong-pitch condition, the pitch of one segment in each pattern was changed to a randomly chosen new pitch, while keeping all other characteristics of that segment (e.g., timbre) unchanged. The randomly chosen pitch for the new segment could keep the contour of the pattern intact, or violate it with equal probability. Each position of a pattern (1–6) could be affected by this manipulation with equal probability in the two distortion conditions—randomly selected from trial to trial. The intensity of the white noise segment was root-mean-square equalized to the rest of the pattern. Figure 1 depicts an example of a train with three patterns and the beginning of the following train.

At the beginning of the first session, ability of the participants to tell whether two melodic phrases are the same or different was measured *via* the melody part of musical ear test (MET) of Wallentin et al. (2010). It contains 52 trials during which two short melodies (comprising of three to eight tones) are played with a tempo of 100 beats per minute, one after the other, with sampled piano sounds. This part of the MET lasts approximately 10 min. In half of the 52 trials, the two melodies are identical. In order to be above-chance level, the participants must score 32 out of 52 trials correctly (= 62%).

During the experimental sessions, the participants were seated in an electrically and acoustically shielded chamber in our laboratory at the Institute of Psychology of Leipzig University. To minimize eye movements, the participants fixated a cross on a computer screen placed behind a window outside the chamber 130 cm from the eyes of the participants. Auditory stimuli were presented binaurally over headphones (Sennheiser HD 25) at an intensity level of approximately 78 dB SPL. The participants were not informed about the roving rule. While listening to the presented sound patterns, the subjects performed a loudness change detection task in order to ensure their attention on the auditory stimulation. Occasionally, the sound patterns were presented with a higher volume (+ 4 dB, five sound patterns = 2% per block) or with a lower volume (–4 dB, five sound patterns = 2% per block). The participants pressed the left button of a response pad as soon as they detected a sound pattern of lower volume and the right button of the response pad as soon as they detected a sound pattern of higher volume. The targets were distributed randomly over each block with the restriction of at least two non-targets in between. After finishing a block, the participants received feedback on their performance (a ratio of hits, interchanged buttons, false alarms, and their mean reaction time). After each block, the subjects had a short break, allowing for movements.

Additionally, at the end of the second experimental session, the participants performed an active pattern change detection task to measure behavioral detectability of pattern changes. In these active blocks, the SOA was prolonged to 1,100 ms for the participants to have sufficient time for solving the task. They were instructed to detect the onset of a new train while ignoring segment distortions. They pressed a button of the response pad as soon as they detected a change to a new sound pattern. After short training, each subject performed one block of each distortion condition, consisting of 200 trials each (8 times train lengths 2, 3, 6, and 12; 16 times train length 1). The order of conditions was counterbalanced over the participants.

### Data Acquisition and Analyses

Electroencephalography data were collected continuously from 64 Ag/AgCl active electrodes. The electrodes were positioned according to the international 10–20 system in a nylon cap. The vertical and horizontal electrooculograms (EOG) were measured with external electrodes placed above and below the right eye and at the outer canthi of both eyes, respectively. As possible offline references, additional electrodes were placed on the tip of the nose and over each mastoid. All electrode signals were (DC) amplified and continuously sampled with a rate of 512 Hz (an anti-aliasing filter with −3 dB at 1/4 of the sampling frequency) by BioSemi Active-Two amplifiers. No high-pass filter was applied online. The BioSemi system uses the common mode sense (CMS) and the driven right leg (DRL) electrode—placed at different sites at the back of the head—to reference the recording to the CMS–DRL ground while minimizing the effect of external noise sources^1^.

Offline, EEG data were re-referenced to the average signal of the two mastoids (Paavilainen et al., 1989; Ritter et al., 1992; Schröger, 1997) and filtered using a 0.5 Hz high pass filter (transition bandwidth of 1 Hz, filter order 1,690) and a 35 Hz low pass filter (transition bandwidth of 10 Hz, filter order 170). Both filters were Kaiser windowed sinc FIR filters (beta = 5.653, stopband attenuation = –60 dB implemented in EEGLAB (Delorme and Makeig, 2004; Widmann et al., 2015). Epochs of 650-ms duration were extracted from the continuous electroencephalography (EEG) time-locked to sound pattern onset. No baseline correction was applied to avoid the introduction of pre-stimulus neuroelectric activity in the baseline period into the post-stimulus waveforms (Urbach and Kutas, 2006). Epochs were sorted for each participant, condition, and stimulus type. Artifactual epochs with a signal range exceeding 100 μV on any recording channel (including EOG channels) were discarded from the analyses. To exclude trials containing artifacts, which are not characterized by extreme amplitude but by noise, we additionally ran a sorted averaging procedure (Muhler and von Specht, 1999; Rahne et al., 2008), during which all epochs of one condition and one participant were sorted according to their noise level (quantified by the root mean square of the voltage and sorted from lowest to highest) and successively entered the average as long as they increase the overall signal-to-noise ratio of the average. Epochs with extreme noise levels, which have a deteriorating effect on the signal to the noise ratio, were excluded. On average, 87 epochs (= 73%) remained for analysis across conditions and the participants. The data of all the participants went into analyses.

Event-related potentials were averaged for deviant sounds (the first pattern of a new train) and for standard sounds (the last corresponding pattern of the preceding train) in each condition. Difference waves were computed by subtracting the standard from the deviant ERPs. To compare ERP effects, in general, between the three conditions, we pooled the deviant and standard ERPs of train lengths 3, 6, and 12 in order to extract a general difference waveform in each condition (general analysis). Train lengths 1 and 2 were not included in the general analysis, because former studies showed that perceptual regularity extraction requires at least two repetitions (i.e., three presentations) of the standard before unexpected complex sound patterns elicit an MMN (Bendixen and Schröger, 2008; Bader et al., 2017). With non-parametric cluster-based permutation tests (Maris and Oostenveld, 2007), we tested for the presence of significant MMN and P3a components in each condition within this general analysis by comparing the whole epoch of the deviant ERP with the standard ERP (α-level for channels and clusters: Monte Carlo *p* < 0.05). About 1,000 permutations were run for each test, and dependent samples *t*-tests quantified the effect. These analyses and the creation of topographical scalp plots were run in FieldTrip (Oostenveld et al., 2011).

For the statistical assessment, amplitude measures were derived from the individual ERPs as the mean signal amplitude in the component time interval for combined conditions (MMN: 200–300 ms after the stimulus onset, P3a: 400–500 ms after the stimulus onset). An estimate of relative slope of each component at the latency for which 75% of the peak amplitude was reached was determined, using the jackknife approach (Miller et al., 1998; Kiesel et al., 2008).

The MMN and P3a amplitude were separately subjected to a repeated measures analysis of variance (ANOVA) with the factors condition (exact, wn, wp), stimulus type (deviant, standard), and train length (1, 2, 3, 6, and 12) as within-subject factors. For the MMN, maximal peak deflections were distributed over different electrodes in central-parietal regions (CPz, Pz, POz). Following a data-driven approach, we focused our MMN analyses on this region with maximal amplitude, and we discuss this rather unexpected topography later (see Discussion). To examine possible topography differences, the factor electrode (CPz, Pz, and POz) was added to the ANOVA. P3a measures were taken from electrode FCz because amplitude deflected maximally at this electrode in all conditions. Three condition-wise repeated measures ANOVAs, with the factors stimulus type (deviant, standard) and train length (1, 2, 3, 6, and 12), and two stimulus-type-wise repeated measures ANOVAs, with the factors condition (exact, wn, and wp) and train length (1, 2, 3, 6, and 12), were run to explore the three-way interactions for MMN and P3a. To investigate in more detail whether and how amplitude of MMN and P3a, as well as the contribution of deviant and standard stimuli to amplitude of both components, changed systematically as a function of train length; *post hoc* logarithmic trend analyses were run for difference waveforms, deviant and standard ERPs, separately. The standardized regression coefficient *r* and its 95% confidence interval (lower and upper bounds) were reported. All ANOVAs generalized eta squared (*η*^2^) served as an estimate of effect size (Bakeman, 2005), and the Greenhouse–Geisser correction was applied when the assumption of sphericity was violated (corrected *df*s were reported). All parametric statistical analyses and the creation of figures were run with RStudio (RStudio. PBC. Version 1.3.1073).

To ensure the participants attended to the auditory stimulation during the EEG session, we analyzed sensitivity *d*′ according to Macmillan and Creelman (2004) to the loudness changes. Sensitivity *d*′ was adjusted for extreme hit or false alarm rates to avoid infinite *d*′ values by adding 0.5 to all response counts (Brown and White, 2005; Hautus and Lee, 2006). Reaction times were measured by calculating the latency between the pattern onset and the key press. Response latencies greater than two SOAs (1,300 ms) were excluded from analysis. To check further for unwanted side effects, the behavioral data in the loudness change detection task were additionally analyzed in repeated measures ANOVA with the factors session (first, second) and condition (exact, wn, and wp).

The active pattern change detection task at the end of the second EEG session was analyzed in terms of the signal detection theory, by extracting an index of sensitivity *d*′ (Macmillan and Creelman, 2004) and correcting it for an extreme hit or false alarm rates by adding 0.5 to all response counts (Brown and White, 2005; Hautus and Lee, 2006). Reaction times were measured by calculating the latency between the pattern onset and the key press. Responses with latencies greater than the SOA (1,100 ms) were excluded from the analysis. To compare the behavioral performance between conditions, repeated measures ANOVAs and Bonferroni-corrected two-tailed *t*-tests were run. Cohen’s *d* was calculated as an estimate of effect size for Student’s *t*-tests. Additionally, hit rates were analyzed *via* repeated measures ANOVA with the factors condition (exact, wn, and wp) and train length (1, 2, 3, 6, and 12) and *via* logarithmic trend analysis to investigate behavioral effects condition-wise and as a function of the number of previous standard repetitions.

## Results

### Behavioral Performance in the MET and in the Loudness Change Detection Task

In the MET, the participants scored, on average, 72% correct (range: 42–88% *SD* = 12%, 17/19 subjects scored above chance). In the loudness change detection task, which participants performed during the EEG recordings, the targets were discriminated with high accuracy. In the first session, averaged sensitivity over the participants (*N* = 19) was *d′* = 3.71 (*SD* = 0.58) in the exact-repetition condition, *d′* = 3.45 (*SD* = 0.64) in the white-noise condition, and *d′* = 3.60 (*SD* = 0.70) in the wrong-pitch condition. In the second session, averaged sensitivity over the participants (*N* = 19) was *d′* = 3.92 (*SD* = 0.52) in the exact-repetition condition, *d′* = 3.80 (*SD* = 0.51) in the white-noise condition, and *d′* = 3.92 (*SD* = 0.58) in the wrong-pitch condition. A repeated measures ANOVA with the factors session (first, second) and condition (exact, wn, and wp) revealed the main effect of session [*F*(1, 18) = 39.17, *p* < 0.001, *η*^2^ = 0.69] but no significant effect of condition [*F*(2, 36) = 3.07, *p* = 0.060, *η*^2^ = 0.15]. No interaction between condition and sessions was found [*F*(2, 36) = 0.52, *p* < 0.60, *η*^2^ = 0.03].

In the first session, reaction times for correctly detected target sounds were *M* = 634 ms (*SD* = 50 ms) in the exact-repetition condition, *M* = 664 ms (*SD* = 53 ms) in the white-noise condition, and *M* = 631 ms (*SD* = 49 ms) in the wrong-pitch condition. In the second session, reaction times for correctly detected target sounds were *M* = 609 ms (*SD* = 59 ms) in the exact-repetition condition, *M* = 628 ms (*SD* = 58 ms) in the white-noise condition, and *M* = 617 ms (*SD* = 54 ms) in the wrong-pitch condition. A two-way repeated measures ANOVA revealed a significant effect of condition [*F*(2, 36) = 13.99, *p* < 0.001, *η*^2^ = 0.04] and of session [*F*(1, 18) = 14.08, *p* = 0.001, *η*^2^ = 0.05]. These main effects were not qualified by an additional interaction [*F*(2, 36) = 1.32, *p* = 0.28, *η*^2^ = 0.01]. Bonferroni-corrected two-tailed *t*-tests showed that, across the sessions, the reaction times in the white-noise condition differed significantly from the exact-repetition condition and the wrong-pitch condition [exact vs. wn: *t*(37) = –3.59, *p* = 0.003 *d* = –1.18; wn vs. wp: *t*(37) = 3.09, *p* = 0.001, *d* = 1.02; exact vs. wp: *t*(37) = –0.43, *p* = 1.000, *d* = –0.14].

The participants showed a high sensitivity in the active pattern change detection task at the end of the EEG recordings in the exact-repetition condition [*d′* = 3.12 (*SD* = 0.77)]. The sensitivity in the white-noise condition was *d′* = 1.90 (*SD* = 0.47) and in the wrong-pitch condition *d′* = 0.96 (*SD* = 0.28). Repeated measures ANOVA comparing the three conditions revealed a significant effect of condition [*F*(0.96, 34.53) = 125.68, *p* < 0.001, *η*^2^ = 0.74]. Bonferroni-corrected two-tailed *t*-tests showed significant differences between each condition pair [exact vs. wn: *t*(18) = 8.47, *p* < 0.001, *d* = 3.99; wn vs. wp: *t*(18) = 10.12, *p* < 0.001, *d* = 4.77; exact vs. wp: *t*(18) = 13.31, *p* < 0.001, *d* = 6.27].

**FIGURE 2:**
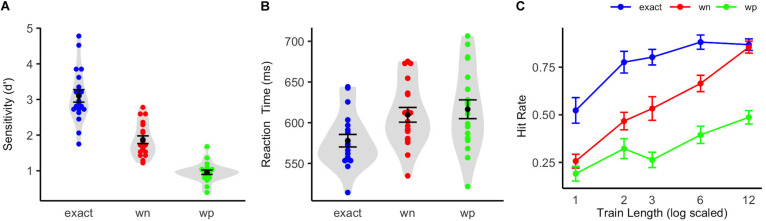
Behavioral performance in the active pattern change detection task. **(A)** Violin plots show participants sensitivity index *d′* when actively detecting pattern changes in the exact-repetition condition (exact: blue), in the white-noise condition (wn: red), and in the wrong-pitch condition (wp: green). **(B)** Violin plots show participants reaction times in the active pattern change detection task. The colored dots indicate the results of each single participant (*N* = 19). The black dots show the mean of all the participants, and whiskers indicate the corresponding standard error of the mean. The shapes (gray) show the distribution of results over all the participants. **(C)** Mean proportion of hits for the exact-repetition condition (exact: blue), the white-noise condition (wn: red), and the wrong-pitch condition (wp: green) on deviant sound patterns as a function of the number of preceding standard sound patterns are shown. Whiskers indicate standard errors of mean.

On average, reaction times for correctly detected target sounds were *M* = 578 ms (*SD* = 34 ms) in the exact-repetition condition, *M* = 610 ms (*SD* = 39 ms) in the white-noise condition, and *M* = 617 ms (*SD* = 50 ms) in the wrong-pitch condition. A one-way repeated measures ANOVA revealed a significant effect of condition [*F*(2, 36) = 12.54, *p* < 0.001, *η*^2^ = 0.15]. Bonferroni-corrected two-tailed *t*-tests revealed significant differences between exact vs. wn: *t*(18) = −3.94, *p* = 0.003, *d* = –1.86 and exact vs. wp: *t*(18) = –3.90, *p* < 0.003, *d* = 1.84. The difference in reaction times between the white noise and the wrong-pitch condition was not significant [*t*(18) = –1.08, *p* = 0.88, *d* = 0.51]. The results of sensitivity and reaction time analysis can be seen in Figures 2A,B.

A repeated measures ANOVA on the hit rates, including the factors condition (exact, wn, and wp) and train length (1, 2, 3, 6, and 12) showed a significant main effect of condition [*F*(2, 36) = 103.98, *p* < 0.001, *η*^2^ = 0.48] and a significant main effect of train length [*F*(4, 72) = 51.36, *p* < 0.001, *η*^2^ = 0.35]. These main effects were qualified by an additional interaction [*F*(8, 144) = 5.11, *p* < 0.001, *η*^2^ = 0.08]. A highly significant logarithmic trend for the train length effect was revealed for each condition [exact: *F*(1, 18) = 22.52, *p* < 0.001, *η*^2^ = 0.56; wn: *F*(1, 18) = 228.30, *p* < 0.001, *η*^2^ = 0.93; wp: *F*(1, 18) = 35.10, p < 0.001, *η*^2^ = 0.66]. Logarithmic regression analyses revealed a steeper increase of the hit rates with increasing train length in the white-noise condition than in the wrong-pitch condition, as confidence intervals did not overlap (wn: *r* = 0.23; CI: 0.19–0.28, wp: *r* = 0.11; CI: 0.07–0.16). A ceiling effect in the exact-repetition condition caused a non-optimal fit of the logarithmic regression and, therefore, an overlap with the confidence interval of the wrong-pitch condition, but not so with the white-noise condition (*r* = 0.13; CI: 0.08–0.18). Mean hit rates are depicted in Figure 2C.

### EEG Data

**FIGURE 3:**
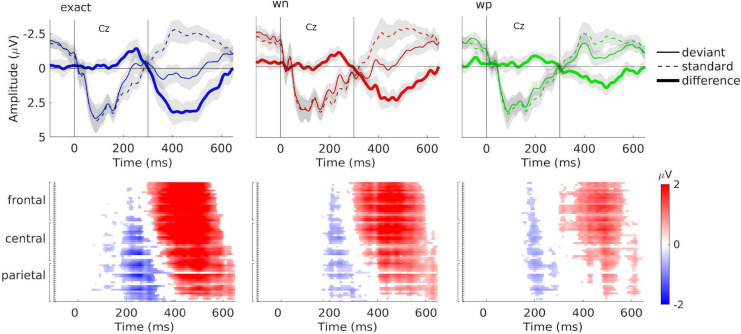
Grand-averaged event-related potentials (ERPs), difference waveforms, and results of the cluster-based permutation test. *Top:* Grand-averaged ERPs at electrode Cz for combined train lengths 3, 6, and 12 in the exact-repetition condition (exact: blue), in the white-noise condition (wn: red), and in the wrong-pitch condition (wp: green). ERPs were elicited by standards (dashed line) and deviants (solid line). Difference waves of the grand-averaged ERPs (deviants minus standards) are shown as thick solid lines. Vertical solid lines indicate the pattern onset at 0 ms and the pattern offset at 300 ms. *Bottom:* Plots are illustrating significant differences (cluster *p* < 0.05) between ERPs to deviants and standards according to cluster-based permutation tests for combined train lengths 3, 6, and 12. Red and blue portions indicate time points/electrodes in which the ERPs to deviants are more positive and negative, respectively. Color brightness indicates the amplitude of the difference. White portions indicate time points/electrodes at which no significant differences were found. Negative deflections (blue) can be seen around 200–300 ms, and positive deflections (red) are found between 300 and 600 ms after the stimulus onset.

**FIGURE 4:**
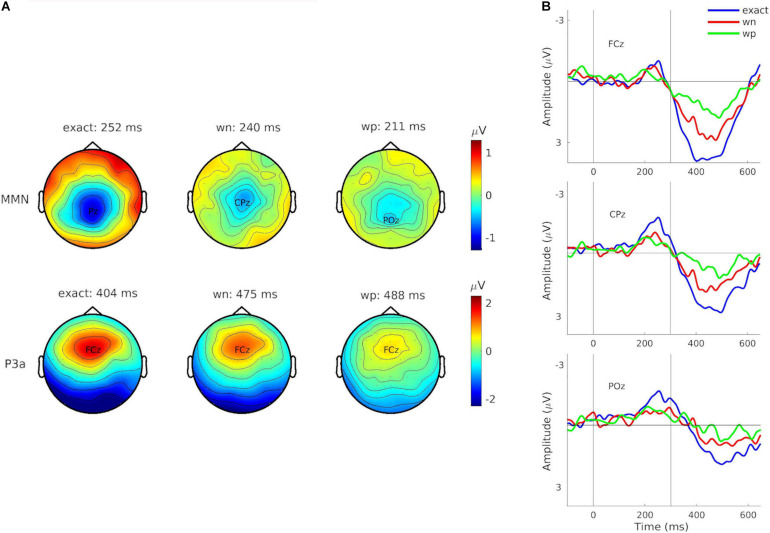
**(A)** Potential topographies of the grand-averaged event-related potential (ERP) differences for combined train lengths 3, 6, and 12 for the components of interest mismatch negativity (MMN) (*top*) and P3a (*bottom*), depending on condition (*left*: exact-repetition condition, exact; *middle*: white-noise condition, wn; *right*: wrong-pitch condition—wp). Topographies show the time point with the maximal amplitude deflections. Corresponding electrodes are highlighted. **(B)** Difference waves of the grand-averaged ERPs (deviants minus standards) for combined train lengths 3, 6, and 12 at electrodes FCz (*top*), CPz (*middle*), and POz (*bottom*). Conditions are indicated by different colors (exact: blue, wn: red, and wp: green). Vertical solid lines indicate the pattern onset at 0 ms and the pattern offset at 300 ms.

The non-parametric cluster-based permutation tests (see Figure 3) showed that grand-averaged difference waveforms (collapsed for train length 3, 6, and 12) elicited negative deflections prior to 300 ms after the stimulus onset in all conditions, likely reflecting MMN, even though its amplitudes were largest at central-parietal and parietal electrodes (exact: Pz: *M* = –1.81 μV at 252 ms; wn: CPz: *M* = –1.01 μV at 240 ms; wp: POz: *M* = –0.91 μV at 211 ms). This negative component was followed by a positive deflection in all conditions, peaking at electrode FCz (exact: *M* = 3.90 μV at 402 ms; wn: *M* = 1.83 μV at 475 ms; wp: *M* = 1.79 μV at 488 ms), reflecting P3a (see Figure 4B).

### MMN Latency

Jackknife estimates of the MMN slope latency of the grand-averaged difference waves (at collapsed electrodes CPz, Pz, and POz), using the relative 75% peak amplitude criterion of the grand-averaged difference waves were *M_*jack*_* = 223 ms (*SD*_*jack*_ = 3.12 ms) for the exact-repetition condition, *M_*jack*_* = 175 ms (*SD_*jack*_* = 14.38 ms) for the white-noise condition and *M_*jack*_* = 194 ms (*SD_*jack*_* = 2.31 ms) for the wrong-pitch condition. A one-way repeated measures ANOVA did not reveal a significant effect of condition: *F*_*adj*_ (1.11, 19.93) = 0.44, *p* = 0.51, *η*^2^ = 0.09.

### P3a Latency

Jackknife estimates of the P3a slope latency at electrode FCz of relative 75% peak amplitude criteria of the difference were *M_*jack*_* = 369 ms (*SD_*jack*_* = 2.46 ms) for the exact-repetition condition, *M_*jack*_* = 402 ms (*SD_*jack*_* = 1.19 ms) for the white-noise condition and *M_*jack*_* = 428 ms (*SD_*jack*_* = 7.08 ms) for the wrong-pitch condition. A one-way repeated measures ANOVA did not reveal a significant condition effect: *F*_*adj*_ (1.19, 21.45) = 2.68, *p* = 0.12, *η*^2^ < 0.001.

### MMN Mean Amplitudes

ERP difference waves and scalp distributions of MMN (and P3a) can be seen in Figure 4.

A four-way repeated measures ANOVA with the factors condition (exact, wn, wp), stimulus type (dev, stand), train length (1, 2, 3, 6, and 12) and electrode (CPz, Pz, and POz) for mean amplitudes in the MMN time window (200 to 300 ms after the stimulus onset) neither revealed a significant 4-way interaction [*F*(6.36, 114.44) = 1.50 *p* = 0.18 *η*^2^ < 0.001] nor any three-way interaction, including the factors electrode and condition [condition × type × electrode: *F*(2.76, 49.64) = 1.05, *p* = 0.38, *η*^2^ < 0.001; condition × train length × electrode: *F*(5.48, 98.59) = 1.12, *p* = 0.36, *η*^2^ < 0.001] that would point to topographical differences between conditions.

Instead, the ANOVA revealed significant main effects [condition: *F*(2, 36) = 17.74, *p* < 0.001, *η*^2^ = 0.05; stimulus type: *F*(1, 18) = 41.47, *p* < 0.001, *η*^2^ = 0.07; train length: *F*(4, 72) = 3.70, *p* = 0.01, *η*^2^ = 0.02] and significant two-way interactions [condition × stimulus type: *F*(2, 36) = 4.37, *p* = 0.02, *η*^2^ = 0.02; condition × train length: *F*(8, 144) = 4.53, *p* < 0.001, *η*^2^ = 0.03; stimulus type × train length: *F*(4, 72) = 26.05, *p* < 0.001, *η*^2^ = 0.11]. All those effects were qualified by a three-way interaction condition × stimulus type × train length: *F*(8, 144) = 2.54, *p* = 0.01, *η*^2^ = 0.03.

**FIGURE 5:**
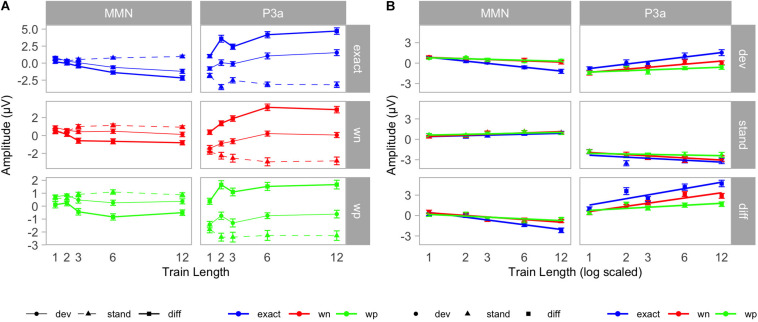
**(A)** Grand-averaged mean amplitudes in the mismatch negativity (MMN) (*left)* and P3a *(right)* time windows. ERPs were elicited by standards (triangles/dashed lines) and deviants (points/solid thin lines). Difference wave amplitudes (deviant minus standard) are shown by rectangles and solid thick lines. Results are plotted condition-wise (*top*: exact/blue, *middle*: wn/red, *bottom*: wp/green). Whiskers denote standard errors of the mean. **(B)** Logarithmic regression analysis. Mean amplitudes in the MMN (*left*) and P3a (*right*) time windows. Results are plotted stimulus-type wise (*top*: deviants/points; *middle*: standards/triangles; *bottom:* differences/rectangle). Conditions are indicated by colors (exact: blue, wn: red, and wp: green). Whiskers denote standard errors of the mean. Train lengths are scaled logarithmically, and trend lines indicate the lines of the best fit.

Subsequently, we first tested whether in each of the three conditions the deviant ERP differed from the standard ERP (main effect of stimulus type) and whether this difference developed with increasing train length (interaction stimulus type × train length). Both the stimulus type effect [exact: *F*(1, 18) = 25.40, *p* < 0.001, *η*^2^ = 0.16; wn: *F*(1, 18) = 5.54, *p* = 0.03, *η*^2^ = 0.03; wp: *F*(1, 18) = 5.76, *p* = 0.03, *η*^2^ = 0.03] and the interaction stimulus type × train length [exact: *F*(4, 72) = 19.25, *p* < 0.001, *η*^2^ = 0.20; wn: *F*(4, 72) = 5.17, *p* = 0.001, *η*^2^ = 0.10; wp: *F*(4, 72) = 4.05, *p* = 0.01, *η*^2^ = 0.06] were significant in each condition. Particularly, the stimulus type × train length interactions resulted from increasingly negative MMN amplitudes of the deviant minus standard difference with train length (see Figure 5A).

To further explore the origin of the condition × stimulus type × train length interaction, we analyzed deviant and standard amplitudes at the centro-parietal electrodes in separate two-way ANOVAs for possible train length effects and interactions with condition. Here, we found for the deviants a significant condition [*F*(2, 36) = 12.03, *p* < 0.001, *η*^2^ = 0.11] and train length effect [*F*(4, 72) = 20.92, *p* < 0.001, *η*^2^ = 0.16] and a significant condition × train length interaction [*F*(8, 144) = 4.97, *p* < 0.001, *η*^2^ = 0.08]. However, for standards, the two-way ANOVA revealed only a train length effect [*F*(4, 72) = 8.05, *p* < 0.001, *η*^2^ = 0.07]. The condition effect [*F*(2, 36) = 2.41, *p* = 0.10, *η*^2^ = 0.01] and the condition × train length interaction [*F*(8, 144) = 1.19, *p* = 0.31, *η*^2^ = 0.02] were not significant. This indicates a similar development of the standard amplitudes with train length independently of condition and a different development of deviant amplitudes with train length in dependence of condition. Furthermore, we observed a significant logarithmic trend for the increase in deviant negativity with increasing train length in all three conditions [exact: *F*(1, 18) = 67.48, *p* < 0.001, *η*^2^ = 0.79; wn: *F*(1, 18) = 13.22, *p* = 0.002, *η*^2^ = 0.42; wp: *F*(1, 18) = 5.20, *p* = 0.04, *η*^2^ = 0.22] (see Table 1). Logarithmic regression analyses revealed a steeper increase in negativity for deviants in the exact-repetition condition (*r* = –0.82; CI: –1.05 to –0.59) than in the white noise (*r* = –0.28; CI: –0.49 to –0.06) and wrong-pitch condition (*r* = –0.20; CI: –0.39 to –0.02) (see Table 1 and Figure 5B).

**TABLE 1:** Logarithmic trend and regression analysis for the main effect of train length on MMN and P3a amplitudes.

	MMN				P3a			
	*F*(1, 18)	*P*	*η*^2^	β (upper bound - lower bound)	*F*(1, 18)	*P*	*η*^2^	β (upper bound - lower bound)
**standards**								
exact	4.82	0.04	0.21	0.19 (−0.00 to 0.38)	11.70	0.00	0.40	−0.41 (−0.79 to −0.03)
wn	14.25	0.00	0.44	0.28 (0.09 to 0.46)	18.03	0.00	0.50	−0.45 (−0.84 to −0.05)
wp	4.12	0.06	0.19	0.16 (−0.03 to 0.35)	1.65	0.22	0.08	−0.14 (−0.48 to 0.21)
**deviants**								
exact	67.48	0.00	0.79	−0.82 (−1.05 to −0.59)	47.90	0.00	0.73	0.95 (0.57 to 1.32)
wn	13.22	0.00	0.42	−0.28 (−0.49 to −0.06)	75.38	0.00	0.81	0.67 (0.38 to 0.96)
wp	5.20	0.04	0.22	−0.20 (−0.39 to −0.02)	6.51	0.02	0.27	0.29 (0.01 to 0.56)
**difference**								
exact	52.36	0.00	0.74	−1.01 (−1.27 to −0.75)	70.57	0.00	0.80	1.35 (0.91 to 1.80)
wn	20.18	0.00	0.53	−0.55 (−0.82 to −0.29)	58.64	0.00	0.77	1.11 (0.79 to 1.44)
wp	7.07	0.02	0.28	−0.36 (−0.61 to −0.12)	7.04	0.02	0.28	0.42 (0.11 to 0.73)

### P3a Mean Amplitudes

A repeated measures ANOVA with the factors condition (exact, wn, and wp), stimulus type (dev, stand) and train length (1, 2, 3, 6, and 12) for mean amplitudes in the P3a time window (400 to 500 ms after the stimulus onset) revealed a significant main effect of condition [*F*(2, 36) = 6.47, *p* = 0.004, *η*^2^ = 0.01]. Furthermore, the main effect of stimulus type [*F*(1, 18) = 122.86, *p* < 0.001, *η*^2^ = 0.36] and the main effect of train length [*F*(4, 72) = 4.64, *p* = 0.002, *η*^2^ = 0.01] were found. The two-way interactions condition × stimulus type [*F*(1.50, 26.97) = 28.57, *p* < 0.001, *η*^2^ = 0.07] and train length × stimulus type [*F*(2.76, 49.70) = 38.45, *p* < 0.001, *η*^2^ = 0.09], as well as the interaction condition × train length [*F*(8, 144) = 2.18, *p* = 0.03, *η*^2^ = 0.01], were significant. However, the main effects and the two-way interactions were qualified by an additional three-way interaction of the factors condition × stimulus type × train length: [*F*(8, 144) = 5.13, *p* < 0.001, *η*^2^ = 0.02].

Two-way repeated measures ANOVAs with the factors stimulus type (dev, stand) and train length (1, 2, 3, 6, and 12) revealed the main effect of stimulus type [exact: *F*(1, 18) = 95.95, *p* < 0.001, *η*^2^ = 0.52; wn: *F*(1, 18) = 86.24, *p* < 0.001, *η*^2^ = 0.31; wp: *F*(1, 18) = 57.71, *p* < 0.001, *η*^2^ = 0.20] and an interaction between stimulus type and train length [exact: *F*(4, 72) = 26.51, *p* < 0.001, *η*^2^ = 0.16; wn: *F*(4, 72) = 20.31, *p* < 0.001, *η*^2^ = 0.11; wp: *F*(4, 72) = 4.09, *p* = 0.005, *η*^2^ = 0.04] for each experimental condition. Particularly, the stimulus type × train length interactions resulted from increasing positive P3a amplitudes of the deviant minus standard difference with train length (see Figure 5A).

To further explore the origin of the condition × stimulus type × train length interaction, we analyzed deviants and standards in separate two-way ANOVAs for possible train length effects and interactions with conditions. Here, we found for the deviants a significant condition [*F*(2, 36) = 24.56, *p* < 0.001, *η*^2^ = 0.15] and train length effect [*F*(4, 72) = 31.84, *p* < 0.001, *η*^2^ = 0.16], which was qualified by a significant condition × train length interaction [*F*(8, 144) = 3.90, *p* < 0.001, *η*^2^ = 0.04]. For standards, the two-way ANOVA revealed a train length effect [*F*(4, 72) = 11.76, *p* < 0.001, *η*^2^ = 0.06] and a condition effect [*F*(2, 36) = 10.07, *p* < 0.001, *η*^2^ = 0.03]. Also, the condition × train length interaction [*F*(8,144) = 3.78, *p* < 0.001, *η*^2^ = 0.02] was significant. Despite significant effects and interactions for deviants and standards, the effects differed between conditions in their strength as *post hoc* logarithmic trend analysis shows (see Table 1). We observed a significant logarithmic trend for the increase in deviant positivity with increasing train length in all three conditions [exact: *F*(1, 18) = 47.90, *p* < 0.001, *η*^2^ = 0.73; wn: *F*(1, 18) = 75.38, *p* < 0.001, *η*^2^ = 0.81; wp: *F*(1, 18) = 6.51, *p* = 0.02, *η*^2^ = 0.27]. Logarithmic regression analyses revealed a steeper increase in positivity for deviants in the exact-repetition condition (*r* = 0.95; CI: 0.57–1.32) compared with the wrong-pitch condition (*r* = 0.29; CI: 0.01–0.56). Confidence intervals in the white-noise condition (*r* = 0.67; CI: 0.38–0.96) overlapped with the increase in the exact-repetition condition and the wrong-pitch condition. For standards, we did not find a significant logarithmic trend in the wrong-pitch condition [*F*(1, 18) = 1.65, *p* = 0.22, *η*^2^ = 0.08] in contrast to the exact-repetition condition [*F*(1, 18) = 11.70, *p* < 0.001, *η*^2^ = 0.40] and the white-noise condition [*F*(1, 18) = 18.03, *p* < 0.001, *η*^2^ = 0.50]. The difference waves of the wrong-pitch condition also developed less steeply than in the exact-repetition condition and in the white-noise condition as confidence intervals did not overlap (exact: *r* = 1.35; CI: 0.91–1.80, wn: *r* = 1.11; CI: 0.79–1.44, wp: *r* = 0.42; CI: 0.11–0.73) (see Table 1 and Figure 5B).

## Discussion

In this study, we investigated the implicit memory formation for repeated auditory objects in situations in which single occurrences of the same object were subject to variability. More specifically, in three conditions, we studied how repetitions of unfamiliar short sound patterns lead to the formation of pattern-specific auditory sensory memory representations when single instances of pattern repetitions are identical (exact) or when they contain small distortions by replacing a segment of the pattern information either by white noise (wn) or by a wrong pitched segment (wp). The participants were not explicitly focusing on the pattern-repetition rule and, instead, performed the task of detecting occasionally occurring loudness changes in the auditory sequence. They performed this task with high accuracy and in the second session of the experiment with increased sensitivity and faster reaction times, likely due to familiarity and learning effects. Sensitivity of the participants to detect loudness changes was not affected by condition. However, the reaction times were significantly slower in the white-noise condition. It is possible the white-noise insertions were stronger distractors from the task, because these segments differed in sound quality from all the other segments of the sound pattern.

As expected, the occurrence of a new sound pattern elicited an MMN and a subsequent P3a; both of which appeared with their typical time course according to previous studies, using an auditory oddball (Alho et al., 1997; Schröger and Wolff, 1998b; Roeber et al., 2003; Debener et al., 2005) and roving-standard paradigms (Cowan et al., 1993; Baldeweg et al., 2004; Bendixen and Schröger, 2008; Garrido et al., 2008). However, the central-parietal distribution of an MMN seems rather untypical, since it can usually be observed at fronto-central recording sites (Näätänen et al., 2001, 2007; Winkler, 2007). We already observed rather posterior MMN topography in a previous study, using similar stimulus material in a comparable experimental procedure (Bader et al., 2017). An explanation for this topography could be the fact that the participants attended the sound sequence (although the pattern changes themselves were task irrelevant). That is, attention might have modulated the otherwise automatic mismatch detection process, potentially allowing for the contribution of an N2b-like component. Yet, even in this case, a more central but not a posterior distribution is expected (e.g., Woods, 1992). Furthermore, the task irrelevance of the pattern changes, and the early latency of the negative peak argues rather against an N2b. Coy et al. (2021) indicate that N2b for task-relevant pattern changes occurs later and can be dissociated from MMN, since, in their data, N2b latency is modulated by the difficulty of the deviant discrimination, whereas MMN latency is not. An alternative explanation could be that the auditory task increased the distracting nature of the deviants (Schröger and Wolff, 1998a). This could evoke a prominent and early P3a in the current paradigm (compared with the more typical passive listening situation). An early emergence of a frontally distributed P3a might partly overlap with the MMN time window and shift the topography toward more posterior sites. Nevertheless, more posterior distributions of the MMN have also been reported in other studies in which the participants watched a silent movie, suggesting that the complexity of the auditory stimuli (e.g., speech and action words) could also result in such atypical MMN topography, indicating activation of a more global network (Shtyrov et al., 2004; Hasting et al., 2007).

### Sensory Memory Trace Formation as Indexed by MMN

The MMM implies that the regularity of repeated complex sound patterns was encoded into a predictive model, and that a change in the overall pattern was detected (Denham and Winkler, 2006; Bendixen et al., 2012; Winkler and Schröger, 2015). This was the case for exact pattern repetitions as well as patterns containing distortions of quality of one segment (white noise) or pitch. We conclude that the auditory system quickly forms pattern representations, even when distortions introduce uncertainty into the implicit learning process. Nevertheless, the amplitude of the MMM was smaller with standard variability, suggesting diminished precision of the predictive model.

Yet MMN amplitude was larger in the condition without distortions compared with the conditions with distortions in the standard patterns. This suggests that the certainty about the repetition regularity must be higher in the exact-repetition condition, which results in more pronounced deviant-related negativity. In particular, MMN amplitudes increased as a function of preceding number of standards, following a logarithmic trend in all conditions, suggesting a fast buildup of pattern representations, particularly with the initial pattern repetitions (see also Baldeweg et al., 2004; Costa-Faidella et al., 2011; Bader et al., 2017). In general, two effects caused the growth in MMN: an increasing positivity for standard ERPs and an increasing negativity for deviant ERPs in the respective MMN time window as a function of train length. The steepness, with which MMN amplitudes grew with increasing train length, was modulated by condition. This effect was mainly driven by the deviant responses, which yielded a steeper growth function in the exact repetition condition than in the conditions containing distortions. The train length effects on standard responses were not modulated by condition, yet, already, their size was smaller than those for deviants.

While change detection was affected by whether distortions were introduced or not, it was not modulated by the type of distortion (noise or wrong pitch information). It seems to be mainly the number of intact segments within the sound patterns, guiding the fundamental process of extracting pattern identity representations as generative predictors under uncertainty, at least on this early processing stage.

### The Role of Exact and Distorted Pitch Code on Evaluation Processes as Indexed by P3a and Behavior in an Active Pattern Change Detection Task

Subsequent to the MMN, a P3a component with typical fronto-central distribution was elicited, and systematic repetition-related modulations of amplitudes were found in all the conditions (see also Bendixen et al., 2007; Horvath et al., 2008; Bader et al., 2017). P3a amplitude increased logarithmically as a function of the number of preceding standard stimuli in all the conditions. This is congruent with studies showing that the P300 amplitude for task-irrelevant deviants is increased, if they occur with lower probability (Squires et al., 1975; Katayama and Polich, 1996) since, in our study, decreased local deviant probability (resulting from longer train lengths) led to an increase of P3a. Overall, P3a magnitude is associated with the degree of novelty and constitutes a marker of the evaluative processing of the contextual novelty (Friedman et al., 2001; Bendixen and Schröger, 2008). Even though attention was focused on a rule-independent task, deviant stimuli likely captured involuntarily attention and were evaluated on the basis of their underlying pattern structure in all conditions.

The growth of P3a as a function of train length resulted from both an increasing positivity for deviant ERPs and an increasing negativity for standard ERPs in the respective P3a time window. For both stimulus types, the growth function was modulated by condition. At the level of the difference waveform, the steepest logarithmic increase in P3a amplitudes with train length was found in the exact-repetition condition. The logarithmic growth of P3a amplitudes in the wrong-pitch condition was distinctly less steep compared with the white noise and the exact-repetition condition. Thus, the growth of P3a activity with increasing number of preceding standards seems to be lowest in the condition with wrong pitch information. Consequently, pattern changes in the context of exact repetitions and white noise distortions might need fewer standard presentations to evaluate deviants as potential novels or targets and to trigger a call for attention of equal strength than pattern changes occurring among repetitions with wrong pitch distortions. Condition differences between P3a growths were partly due to the processing of the standard events. In the wrong-pitch condition, standard responses in the P3a time window did not show a logarithmic repetition-related modulation as opposed to the exact repetition and the white-noise-distortion condition. The amplitude decreases with the number of repetitions seems relatively flat, but confidence intervals overlapped with the exact repetition and the white-noise-distortion condition. Overall, the processing of deviating events had a stronger contribution to the condition differences on the P3a level (similar to what we found for the MMN). In the exact-repetition condition, the logarithmic trend of systematic amplitude modulations was much steeper than in the wrong-pitch condition.

Despite the pronounced differences in P3a amplitude, estimated component latencies were not affected by our experimental manipulation. The time needed to internally evaluate pattern identity seems not higher with noise or wrong pitch insertions. Here, the auditory system seems equally quick in classifying the stimuli, in evaluating the novelty of the deviance, and in automatically orienting attention toward the task-irrelevant novel patterns (Polich, 2007; Horvath et al., 2008; Wetzel et al., 2013; Winkler and Schröger, 2015).

The behavioral performance in the active pattern change detection task showed highest accuracy in the exact-repetition condition, less accuracy in the white-noise condition, and least accuracy in the wrong pitch segment condition. The development of the hit rates as a function of train length also followed a logarithmic trend in all conditions with the hit rates in the exact-repetition condition, developing quickly toward a ceiling effect and most slowly in the wrong-pitch condition, mirroring the pattern of results at the level of P3a.

### Effects of the Different Types of Distortion at the Levels of Sensory Memory Formation and Contextual Stimulus Evaluation

Effects in the MMN time range did not distinguish between the two types of distortions that we introduced in the sequences. In both cases, when a pattern segment was occasionally replaced by white noise or by a differently pitched segment, the typical negativity observed for deviant responses grew less steeply compared with the condition with exact pattern repetitions. As the portion of matching pattern segments was similar for both conditions (five out of six), one could speculate that, at this early processing stage, the strength of regularity encoding mainly depends on the probability of matching information in spectrotemporal space. A previous study revealed that object representations can be retrieved from repetitions embedded in different backgrounds and suggested the correlation of input spectrograms between several non-perfect repetitions as a potential mechanism to achieve this (McDermott et al., 2011).

In contrast to the MMN findings, the type of distortion (white noise or wrong pitch information) seems to have a greater impact on evaluation processes at the stage of P3a. Here, we observed a clear disadvantage for the wrong pitch compared with the exact-repetition condition, whereas the response pattern in the white-noise condition resembled that of the exact-repetition condition. This suggests that the higher cognitive evaluation process and the attentional switch toward a deviant pattern are not substantially impaired in the white-noise condition. This could be explained by processes related to stream segregation or perceptual filling-in. Firstly, the white noise segments differed substantially from the other five segments in their spectral composition, leading to a vastly different timbre percept. This could have assisted the segregation of pattern and distracter information, allowing the later stages of evaluating a newly incoming pattern to be less disturbed. Secondly, white noise insertions could even lead to a partial restoration of the perceptual continuity of an intact standard pattern (Warren et al., 1997, 1988), particularly since the pitch forming spectral components of the replaced pattern segment can physically be found in the 50-ms white noise segment. This could explain similar processing of standards and deviants in the exact and the white-noise condition. The processing of wrong pitch information might have prevented segregation and restoration processes. Here, the similar timbre of the distorted segment promoted the percept of a continuous sound pattern, and a segregation mechanism cannot take an effect. Also, the wrong pitch information is simply misleading, and there is no option for a perceptual restoration of erased or ambiguous information. This might have led to the greater disadvantage for the wrong-pitch condition and is compatible with explanations of failed segregation in informational masking studies (Kidd et al., 2008).

Overall, our two types of manipulations might remind of energetic and informational masking. During energetic masking, the processing of a sensory event is degraded typically by a noise masker, where, mainly, the energy relation between the signal and the masker determines their separability and the amount of difficulty in perceiving the signal (Muller-Gass et al., 2001; Darwin, 2008; Moore, 2008; Wilson et al., 2012). During informational masking, it is, additionally, the similarity (either the acoustic or the semantic similarity) between the target and masker sound that deteriorates segregation and perception of the signal sound, such as when a speech signal is masked by speech (Dirks and Bower, 1969; Durlach et al., 2003; Scott et al., 2004; Gutschalk et al., 2008; Kidd et al., 2008; Mattys et al., 2012). In masking studies, it has been found that irrelevant information is easier to suppress when energetic masking is dominant, thus facilitating the processing of relevant sensory information (Lidestam et al., 2014). In contrast, when informational masking is dominant, a widespread general attentional network is activated to distinguish distracter and target information (Szalárdy et al., 2019). In our study, the wrong pitch segment is due to its spectral profile acoustically highly similar to the pattern elements themselves. The uncertainty about which are the five relevant (intact) segments of the tonal pattern is maximally high, and segregation between the true pattern and the replaced distracting element will not be as easy as in the white-noise condition. In that sense, replacing valid pattern information by white noise or by wrong pitch information could lead to distinct forms of interference that might be resolved at different processing levels and even distinguishable neural circuits (Scott et al., 2004).

## Conclusion

In the present study, we demonstrated that the auditory system is able to form pattern representations and predictions even in the context of uncertainty. MMN and P3a were elicited in response to deviants in all conditions at similar latency estimations. Independent of distorted segments, an implicit and automatic buildup of regularity representations and deviance detection, with a following call for attention toward the stimuli, can be assumed. However, we found a general advantage for the exact-repetition condition over the two distorted repetition conditions. This is shown by steeper logarithmic amplitude changes on MMN and the P3a level, with increasing number of repetitions, as well as in the behavioral performance in the active pattern change detection task. The processing on the MMN level does not seem to differentiate between the qualities of the distortions but reacts to general (mis-)matching statistics between the sound patterns. At the level of P3a, we observed an influence of the type of distortion. On this processing stage, white-noise distortions may not have impeded stimulus processing to such an extent as wrong pitch distortions did. This is evidenced by the distinct degradation of the P3a, which also goes along with the behavioral findings in the active pattern change detection task. Additionally, our results revealed that deviant processing is more affected by our experimental manipulations compared with standard processing.

To sum up, our findings indicate that the auditory system is able to quickly extract regularities and generate reliable predictions, even when the to-be-extracted patterns contain distortions. However, higher cognitive processing and the involvement of an attentional network might give the basis for the subtler evaluation of the acoustic input with regard to the type of distortion. The segregation between informative pattern segments and the distorting element, as well as the accessibility of a possible interpolation mechanism (like the one discussed for the continuity illusion), might explain the facilitated processing of white noise compared with wrong-pitch distortions on later processing stages that govern the elicitation of P3a and guide behavioral responses.

## Data Availability Statement

The raw data supporting the conclusions of this article will be made available by the authors, without undue reservation.

## Ethics Statement

This studies involving human participants were reviewed and approved by the Ethics Committee of the University of Leipzig, Germany. The patients/participants provided their written informed consent to participate in this study.

## Author Contributions

SG and ES: conceptualization, supervision, and resources. MB: data curation, visualization, and writing—original draft. MB and SG: formal analysis, investigation, software, and project administration. SG: funding acquisition. SG, ES, and MB: methodology, writing, review, and editing, and validation. All authors contributed to the article and approved the submitted version.

## Conflict of Interest

The authors declare that the research was conducted in the absence of any commercial or financial relationships that could be construed as a potential conflict of interest.

## Footnotes

1 https://www.biosemi.com/faq/cms&drl.htm
